# Pine trees structure plant biodiversity patterns in savannas

**DOI:** 10.1002/ece3.70021

**Published:** 2024-07-17

**Authors:** Raelene M. Crandall, Yingen M. Chew, Jennifer M. Fill, Jesse K. Kreye, J. Morgan Varner, Leda N. Kobziar

**Affiliations:** ^1^ School of Forest Fisheries and Geomatics Sciences University of Florida Gainesville Florida USA; ^2^ Department of Ecosystem Science & Management Pennsylvania State University University Park Pennsylvania USA; ^3^ Tall Timbers Research Station Tallahassee Florida USA; ^4^ Department of Forest Rangeland and Fire Sciences, College of Natural Resources University of Idaho Coeur d'Alene Idaho USA

**Keywords:** canopy cover, flatwoods (mesic), longleaf pine (*Pinus palustris*), prescribed fire, sandhills (xeric), spatial, species richness, turnover

## Abstract

Overstory trees serve multiple functions in grassy savannas. Past research has shown that understory species can vary along gradients of canopy cover and basal area in savannas. This variation is frequently associated with light availability but could also be related to other mechanisms, such as heterogeneity in soil and litter depth and fire intensity. Several savanna studies have found differences in understory plant functional groups within the local environment near trees versus away from them in canopy openings. Although small‐scale variation is known to be high in southeastern U.S. pine savannas, patterns in understory species diversity have not been examined at the scale of individual overstory pine trees in this system. We conducted an observational study of the relationship between understory plant communities and proximity to individual pine trees in xeric and mesic pine savannas in frequently burned sites (1–3 year intervals). We recorded the plant community composition in plots adjacent to tree boles (basal) or outside crown driplines (open). Within each environment, raw species richness was significantly greater in open locations, where light transmittance was greater. In contrast, rarified species richness did not differ. Multivariate analyses showed that community composition differed significantly between basal and open plots. One native, woody species in each environment, *Serenoa repens* (W. Bartram) Small in mesic and *Diospyros virginiana* L. in xeric, was more abundant in basal plots. In mesic environments, eight species had greater occurrence in open plots. In xeric environments, four understory forbs were more abundant in open plots. Our results support previous research indicating that individual pine trees are associated with significant variation in understory vegetation in pine savannas.

## Graphical abstract

Because patterns and processes in pine savannas are scale‐dependent, individual trees could influence at small scales that are not necessarily evident at larger scales and vice versa. Overstory–understory structure shaped by frequent fires likely encourages niche selectivity and deterministic assemblage of plant communities, increasing diversity at larger scales.

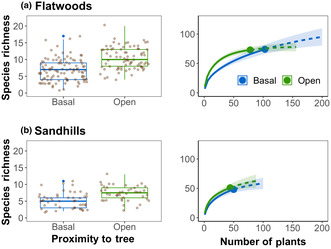

## 1 INTRODUCTION

The presence of trees in savannas contributes to their functional distinction from grasslands. Although both grasslands and savannas feature an herbaceous understory where C4 grasses are common, trees in savannas structurally differentiate these systems and influence ecosystem function (Ratnam et al., 2011; Stahlheber et al., 2015; Veldman et al., 2013). For example, in miombo woodlands of southern Africa, tree species diversity is positively correlated with aboveground carbon stocks (McNicol et al., 2018). In oak savannas of Midwestern North America, thresholds in tree basal area, canopy cover, and fire return interval are related to the amount of herbaceous understory cover (Abella et al., 2023). Herbaceous species richness in savannas of southeastern North America is lower near trees than in openings (Brewer, 1998; Platt et al., 2006). Indeed, overstory–understory interactions are a defining feature of the characteristics and dynamics of savannas worldwide (Ratnam et al., 2011; Scholes & Archer, 1997; Veldman et al., 2014). Savanna trees are also habitat for threatened and endangered birds, mammals, and reptiles (Penton et al., 2021; Zwicker & Walters, 1999). Thus, although savannas are often grouped with grasslands as “grassy biomes” (Bond & Parr, 2010), they should also be considered separately, given the importance of their overstory component.

In southeastern U.S. pine savannas, overstory pine trees have strong relationships with plant communities. Tree density and canopy cover are inversely related to pollinator abundance and are associated with spatial heterogeneity of understory plants across the landscape (McGuire et al., 2001; Mugnani et al., 2019; Odanaka et al., 2020; Platt et al., 2006). Light availability is a key pathway for overstory associations with understory biodiversity in savannas and woodlands (Brewer, 1998; Pilon et al., 2021; Rossatto et al., 2018; Scholes & Archer, 1997), and the pine tree canopy creates heterogeneity in the understory light environment that affects plant dynamics such as seedling establishment (McGuire et al., 2001). For example, Platt et al. (2006) found greater species richness and different species composition in large pine canopy openings with greater light transmittance. At a local level, however, individual trees could differentiate understory plant diversity patterns from those in open patches away from trees (e.g., Johnson et al., 2021; Stahlheber et al., 2015). By influencing fire intensity and severity, for example, shed pine needle fuels can indirectly affect understory plant regrowth and survival near the bases of trees (Ellair & Platt, 2013; Mugnani et al., 2019; Platt et al., 2016; Thaxton & Platt, 2006; Varner et al., 2022). In addition to light availability, other mechanisms for variation near and away from tree boles include soil depth relationships with plant rooting depth (Beck & Givnish, 2021) and litter depth effects on seed germination (Xiong & Nilsson, 1999). Thus, individual pine trees can play a direct role in the structure and dynamics of pine savanna communities.

Our objective was to investigate patterns of understory plant species presence and communities with proximity to the stem and canopy of individual pine trees in frequently burned xeric and mesic pine savannas. We sampled plant community composition under and outside of pine tree canopies in pine stands that have burned with 1–3‐year intervals for more than 40 years. Given previous research in analogous environments, we expected to find differences in plant community composition between under and outside of tree canopies (e.g., McGuire et al., 2001; Pilon et al., 2021; Stahlheber et al., 2015). The heterogeneity associated locally with individual trees could explain a portion of the high diversity reported in savannas worldwide (Mishra & Young, 2020).

## 2 METHODS

### 2.1 Study area

This study was conducted in two pine savanna environments that have been frequently and recurrently burned for over 40 years. The mesic flatwoods and xeric sandhills sites were located in Austin Cary Forest (29°44′ N, −82°14′ W) and Ordway‐Swisher Biological Station (29°40′ N, −81°74′ W), respectively. The two sites, owned and operated by the University of Florida, were approximately 20 km apart in northern Florida, USA. Within these environments, we examined multiple burn units within 2.5 km of each other, two in xeric sandhills and four in mesic flatwoods. Sites were chosen to allow for comparisons within environment types with contrasting soil characteristics and moisture profiles: mesic flatwoods with mostly Spodosols and xeric sandhills dominated by Entisols. Both environments are characterized by an open canopy of tall longleaf (*Pinus palustris* Mill.) and slash (*Pinus elliottii* Engelm.) pines and a diverse ground layer of low shrubs, grasses, and forbs (Florida Natural Areas Inventory, 2010).

Mesic flatwoods and xeric sandhills pine savannas differ in their species richness and share few species. Flatwoods typically have slightly higher species richness than sandhills, with an average of 8.40 versus 6.25 species in the 1 × 1 m plots we sampled. Flatwoods were dominated in the understory by saw palmetto (*Serenoa repens* (W. Bartram) Small), fetterbush (*Lyonia lucida* (Lam.) K. Koch), dwarf huckleberry (*Gaylussacia dumosa* (Andrews) A.Gray), shiny blueberry (*Vaccinium myrsinites* Lam.), and diverse flowering perennials. In contrast, sandhills had sparser vegetation, often with patches of bare sand, and were dominated by wiregrass (*Aristida beyrichiana* Michx.) with occasional shrubby oaks (mainly *Quercus laevis* Walter). Most woody species in these pine savanna environments, except for pines (*Pinus* sp. L.), are short in stature and remain a component of the understory when fires are frequent and recurrent. Wiregrass was the only species that dominated both pine savanna types.

Previous research was conducted in our study sites using many of the same trees. Kreye et al. (2020) found no differences in maximum temperature or duration of soil heating adjacent to pine tree stems compared to outside the driplines of pine trees in frequently burned sites. In addition, Kobziar et al. (2019) found that fires in these frequently burned sites did not consume a significant amount of litter, although fuel loads were minimal prior to fires. The fires also had little effect on soil respiration, whether adjacent to or outside the driplines of pines (Kobziar et al., 2019). These results contrast what would be expected in long, unburned pine savannas that have undergone mesophication, with a closed overstory and significant litter and duff accumulation.

### 2.2 Sampling

We installed sampling plots around haphazardly selected trees. Dominant or co‐dominant mature trees with no apparent signs of stress or injury (i.e., dead foliage or branches, visible signs of insect or fungal pathogen) and diameter at breast height between 25 and 40 cm were randomly selected from multiple locations within management units, at least 3 m from a boundary to avoid edge influences. Most of the trees were selected from 1‐hectare areas within the management units as part of a larger study that included installing thermocouple networks. Although this limited the distances between trees to the length of the thermocouple wires, all selected trees were at least 5 m from another selected tree (Kobziar et al., 2019). From the central point radiating outward, each tree that met the criteria and did not have a canopy that completely overlapped with another tree was selected. Additional trees were added to increase replication for community sampling. These trees were randomly selected using the same criteria but had greater distances between them.

To measure community composition and light transmittance, we installed 3–4 plots around each of the haphazardly selected pines. Given the variation around each tree, we installed multiple plots. Two 1 × 1 m plots were positioned adjacent to the base of each pine, with the first plot at each tree randomly located and the second plot on the opposite side of the tree at a 180‐degree angle from the first plot. We hereafter refer to these as “basal” plots. Plots on either side of trees capture the within‐tree variation, such as the structure of longleaf pines, which ranges from symmetrical to those with the majority of branches and needles on one side of a tree. In addition, different sides of trees experience shading during different times of the day. Two additional plots, if possible, were established at the same angles as the basal plots but positioned just beyond the pine's crown dripline, hereafter “open” locations. The number of plots sampled varied by treatment: 39 and 51 open and basal plots in flatwoods, respectively, and 22 and 24 open and basal plots in sandhills, respectively. There are fewer open plots because if tree canopies overlapped on one side, there were no areas beyond the dripline where we could install a second open plot.

During the fall of 2017, we quantified the light transmittance through the canopy using hemispherical photography. Photographs were taken at each plot (1 m above ground level) with a fish‐eye lens during cloudy conditions. We used Gap Light Analyzer software (version 2.0 Simon Fraser University, Burnaby, BC, Canada) to quantify the percent light transmittance from the photos (Frazer & Canham, 1999).

Units in the mesic flatwoods were burned on April 10, 2017, and units in the xeric sandhills were burned on June 20–23, 2017. We acknowledge that using only one flatwoods and one sandhills site may cause a pseudoreplication issue, but sampled trees were selected from multiple burn units to capture the natural variation expected in these sites. See Kreye et al. (2020) for weather and fire behavior during experimental burns. We measured the composition of understory plant species in each plot during the fall of 2017, approximately 6 months after the prescribed fires. In each plot, the presence of every plant was recorded to species (or morphotype when seedlings or small resprouts could not be identified). We revisited plots during 2018, 18 months post‐fire, to identify unknown plants and to ensure we documented any species with a delayed response to fire. During preliminary analyses, we detected no differences in species composition between 2017 and 2018. Thus, only the 2017 data were analyzed. For all analyses, flatwoods and sandhills were analyzed separately, given they do not share many of the same species. Presence/absence was used to calculate raw and rarified species richness and to examine differences in community composition.

### 2.3 Analyses

Percent light transmittance and raw species richness were analyzed using mixed effects models with a Gaussian fit (*lmer* function in “lme4” R package; Bates et al., 2015). The unit of replication was plot (coded as a factor) within tree, and all analyses were conducted at this level. To determine the error structure, we first tested whether there was significantly greater variance for plots nested within trees or for plots between trees (Meier, 2022). Because there was little variance for plots nested within trees, we only included random slopes for trees in each model. Model assumptions (i.e., dispersion and zero inflation) were tested using the “DHARMa” R package (Hartig, 2020). In addition to raw species richness, we calculated rarefaction curves for basal and open plots in each environment using the “iNEXT” R package, indicating the datatype = “incidence_raw” (Hsieh et al., 2020). The diversity estimates for the rarefied and extrapolated samples are calculated up to the data's maximum sample size, or in this instance, number of plants per sample location (i.e., open vs. basal), after which they are extrapolated (Hsieh et al., 2020). Confidence intervals were compared to determine whether significant differences in rarefied species richness existed between basal and open plots for each environment (Gotelli & Chao, 2012).

We determined whether plant community composition differed between basal and open locations using plant species presence/absence data. First, the *metaMDS* function in the “vegan” R package was used on Jaccard distance matrices to visualize plots in ordination space (flatwoods: *k* = 3, stress = 0.16; sandhills: *k* = 3, stress = 0.19; Oksanen et al., 2019). Jaccard distance is typically used for presence/absence data. The *stat_ellipse* function in the “ggplot2” R package was used to draw ellipses representing the 95% confidence level for the multivariate *t*‐distribution (Wickham, 2016). Next, we checked whether the dispersions (i.e., variances) of distance values in basal and open locations differed using the *betadisper* function in the “vegan” R package (Oksanen et al., 2019). An ANOVA indicated that dispersion among groups was heterogeneous for sandhills (*p* = .009) and flatwoods (*p* = .023) pine savannas, so we proceeded by conducting permanovas with 999 permutations using the *adonis2* function in the “vegan” R package to test for significant differences in community composition between basal and open locations (Oksanen et al., 2019).

To examine species‐level differences between locations (open vs. basal), we used multivariate linear models with a matrix of species presence (i.e., binomial distribution) by plot as the response variable and location as the predictor variable. We used the *manyglm* function in the “mvabund” R package to fit Generalized Linear Models (GLMs) for the presence/absence data (Wang et al., 2019). We set p.uni = “adjusted” to specify that univariate *p*‐values should be adjusted for multiple testing, using a stepdown resampling procedure. Next, we plotted the Smyth‐Dunn residuals against the linear predictor values (Wang et al., 2019) and did not observe a fan shape, indicating the data have an approximate normal distribution. The *anova.manyglm* function in “mvabund” was used to test the individual (i.e., univariate) species responses to open versus basal locations and compute an analysis of deviance table for the multivariate GLMs (Wang et al., 2019). Within the model, we specified that rows of the matrix (i.e., plots) should be resampled, providing a better approximation of the true significance level. All analyses were performed in R version 3.6.0 (R Core Team, 2022).

## 3 RESULTS

**FIGURE 1:**
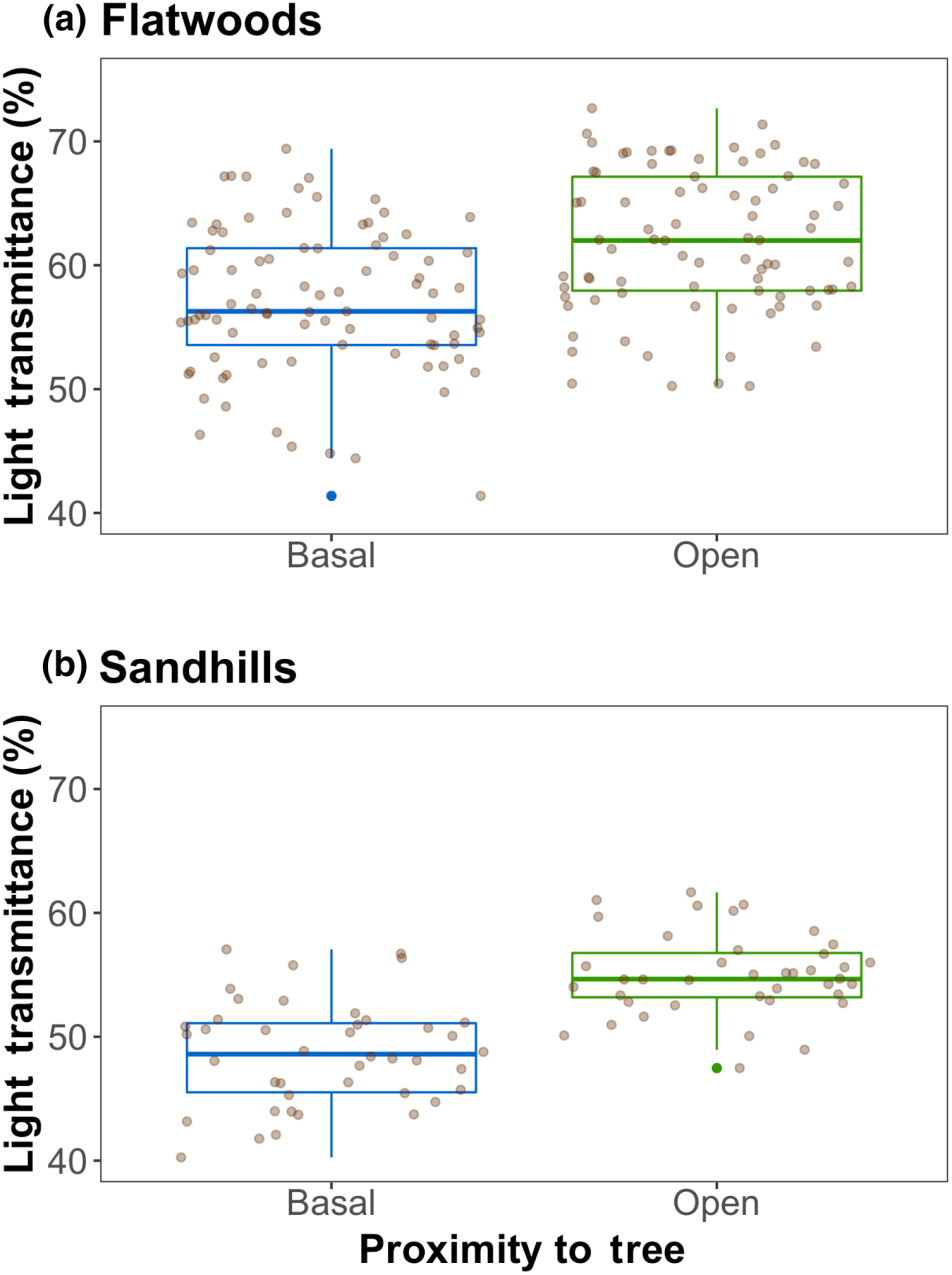
Boxplot with median and 95% confidence intervals of percent light transmittance measured 1 m above plots located adjacent to pine trees (basal) or outside of their driplines (open) in mesic flatwoods (a) and xeric sandhills (b) pine savannas.

In both mesic flatwoods and xeric sandhills, percent light transmittance was lower in basal than open plots as expected. The percent light transmittance was significantly lower in basal plots than in the “open” plots outside of pine tree driplines (both *p* < .001; Figure 1a,b). Overall, percent light transmittance was between 40% and 75% for all plots, indicating that some light reached the savanna ground layer regardless of proximity to a pine tree stem or canopy. On average, light transmittance was 11.5% lower in sandhills and 7.7% lower in flatwoods in basal compared to open locations.

In total, we made 2113 plant observations of 130 species in plots across both environments. Of these, 32 species (24.6% of total species), which totaled 126 observations (6.2% of total observations), were classified as unknown despite multiple efforts to resolve the identifications. In most cases, the 32 species were too immature to key. Because these unknown plants (i.e., observations) had distinct morphological characteristics, we were able to group them into morphotypes confidently. Regardless, none of the morphotypes were significant in any analyses we conducted.

**FIGURE 2:**
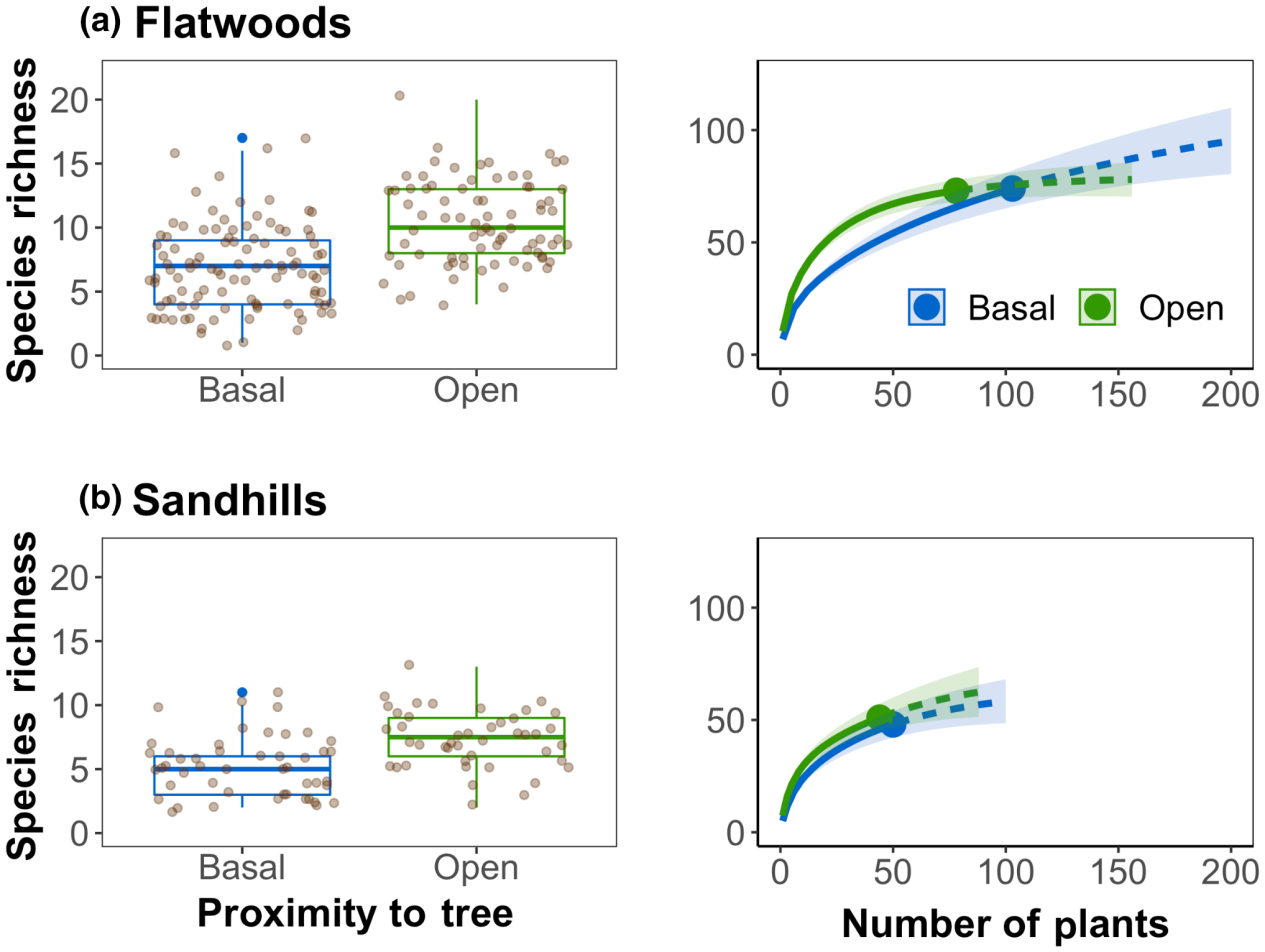
On the left are boxplots of the raw number of species (circles) and standard error (bars) in 1 × 1 m plots located adjacent to pine trees (basal) or outside of their driplines (open) in mesic flatwoods (a) and xeric sandhills (b) pine savannas. On the right are rarefaction (solid lines) and extrapolation (dashed line segments) sampling curves with 95% confidence intervals (shaded areas) for species richness adjacent to pine trees and outside their driplines.

We found contrasting results for differences in plant species richness in open and basal plots based on whether we used raw or rarified species richness. When analyzing species richness using a mixed effects model, we found significantly more species in the open than in basal plots in both sandhills and flatwoods environments (both *p* < .0001; Figure 2a,b). On average, raw species richness was 30.0% lower in sandhills and 34.2% lower in flatwoods in basal compared to open locations. This trend was not observed in species rarefaction curves that estimate species richness (Figure 2). Estimated species richness from rarefaction in flatwoods pine savannas in basal and open plots (with 95% confidence intervals) were 28.0 (21.0, 35.0) and 20.4 (16.2, 24.6), and in sandhills pine savannas in basal and open plots were 96.1 (69.5, 122.7) and 69.8 (64.3, 75.3). Due to the overlapping confidence intervals, there were no differences in species richness when rarefaction was considered.

**FIGURE 3:**
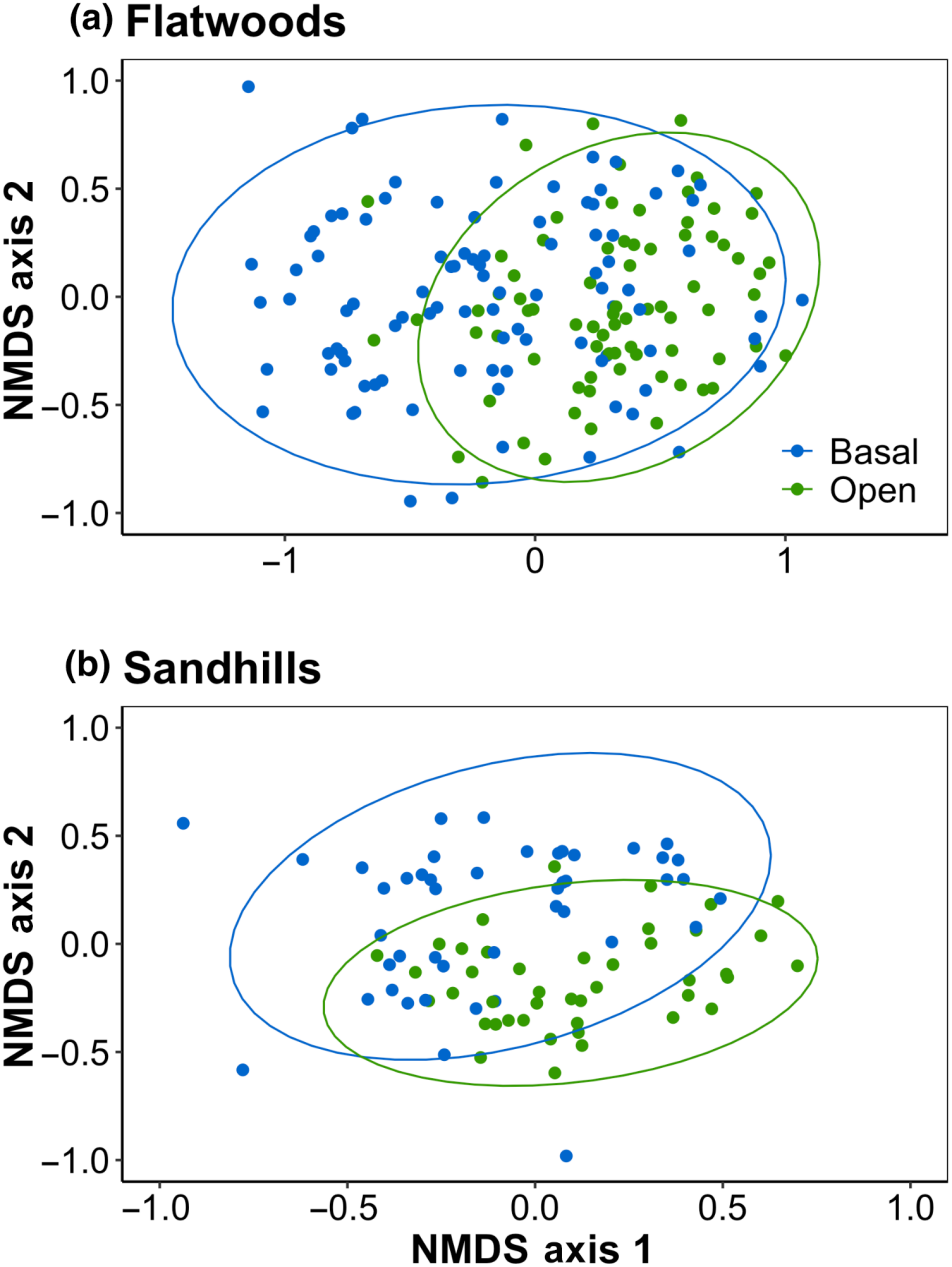
Multivariate analysis of plant community presence/absence data in mesic flatwoods (a) and xeric sandhills (b) pine savannas. The ellipses show the 95% confidence level for the multivariate *t*‐distribution between community composition in basal and open plots combined across fire histories.

**FIGURE 4:**
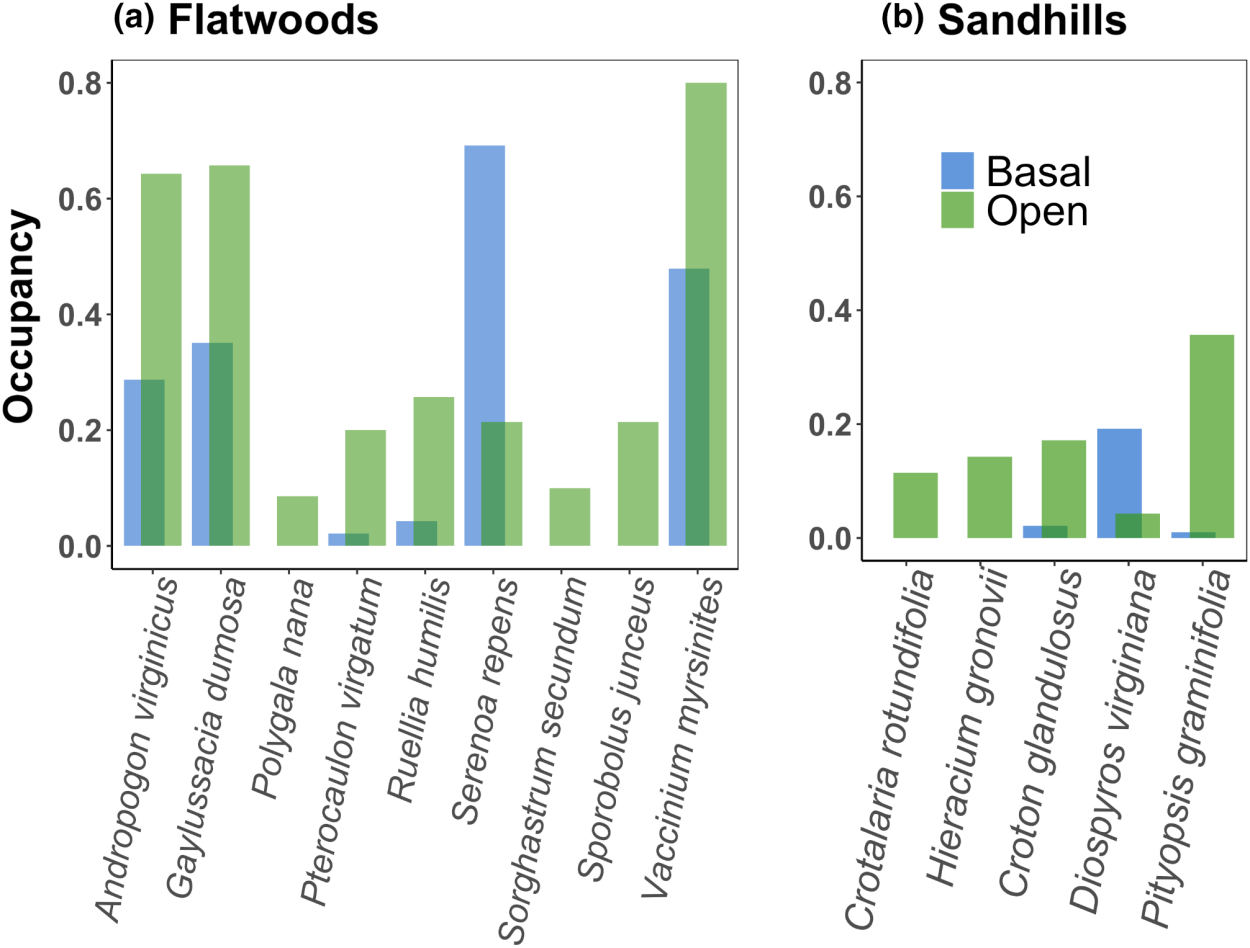
Barplot of occupancies in 1 × 1 m plots located adjacent to pine trees (basal) or outside of their driplines (open) in mesic flatwoods (a) and xeric sandhills (b) pine savannas. *Vaccinium myrsinites* and *Gaylussacia dumosa* are ericaceous shrubs, *Diospyros virginiana* is a small deciduous tree, and *Serenoa repens* is an understory palm. All of these woody species remain in the pine savanna understory, provided fires are recurrent and frequent. The remaining species are understory grasses and forbs. All species listed are native.

In addition to differences in species richness, our multivariate analyses showed that community composition differed between basal and open plots (*p* < .001 for both environments; Figure 3a,b). Two woody species, an understory palm (*Serenoa repens* (W.Bartram) Small) in flatwoods and a small deciduous tree (*Diospyros virginiana* L.) in sandhills, were more dominant in basal plots (*p*unadj. <.005 level; Figure 4a,b). In mesic flatwoods pine savannas, eight native species were more likely to occur in the open as compared to basal plots (Figure 4a). These included two ericaceous shrubs (*Gaylussacia dumosa* (Andrews) Torr. & A. Gray and *Vaccinium myrsinites* Lam.), a small deciduous tree (*Diospyros viriginiana* L.), three grasses (*Andropogon virginicus* L., *Sorghastrum secundum* (Elliott) Nash, and *Sporobolus junceus* (P. Beauv.) Kunth), and three understory forbs (*Polygala nana* (Michx.) DC., *Pterocaulon virgatum* (L.) DC., and *Ruellia humilis* Nutt.). In mesic flatwoods, four native, understory forbs were more abundant in open as compared to basal plots, including *Crotalaria rotundifolia* J. F. Gmel., *Hieracium gronovii* L., *Croton glandulosus* L., and *Pityopsis graminifolia* (Michx.) Nutt. (Figure 4b).

## 4 DISCUSSION

Individual pine trees are associated with significant heterogeneity in understory plant species composition in pine savanna flatwoods and sandhills. Most species that were significant in our analyses had higher abundances in open plots where there was greater light availability, which has been suggested as a key mechanism for differences in vegetation composition in other similar studies (e.g., Brewer, 1998; Lavoie et al., 2012). Although previous research at these sites did not detect differences in some fire‐related characteristics of basal and open locations after one fire (Kreye et al., 2020), the occurrence of certain species close to and away from pines could reflect the long‐term fire history at each point (Brewer, 2023). In addition, they could result from spatial patterns of pine litter deposition, which has been shown to vary with time since fire, decomposition rate, stand structure, and direction of prevailing winds (Blaydes et al., 2023; Sánchez‐López et al., 2023). Fine‐scale fuel heterogeneity results in spatial variation in plant survival and establishment through aboveground fire intensity or soil heating (Platt et al., 2016; Kennard & Outcalt 2006), which ultimately manifests in differential community assembly over time (Mugnani et al., 2019; Robertson et al., 2019). Other potential mechanisms for differences in composition near and away from pines include spatial water and nutrient availability (Alexander et al., 2021). In restoration sites, for example, soil carbon and nitrogen have been found to be higher near pine trees than at several meters from trees (Lavoie et al., 2012).

Although we found that community composition differed between open and basal plots in both environments, species richness results depended on whether raw or rarified species counts were used. Like species composition differences, lower raw species richness beneath the crown could be related to lower light transmittance, with species in basal plots having a greater shade tolerance or those in open plots requiring more light (Leach & Givnish, 1999). Brewer (1998) also found lower herbaceous species richness near (<2 m away) individual slash pine (*Pinus elliottii*) trees and suggested that lower light transmittance was likely an important mechanism. Alternatively, higher light levels may also result in more occurrences (Myers & Harms, 2009; Schnitzer & Carson, 2001; Young & Koerner, 2022). This likely explains why we found no differences in species richness when we used rarefied calculations.

Our study lends support to the significance of individual trees for local‐scale patterns in diversity and ecosystem function. Individual trees provide habitat for wildlife (Kaiser et al., 2020; Penton et al., 2021), cycle nutrients (Kellman et al., 1985; Kellman & Miyanishi, 1982; Rhoades, 1996), structure understory competitive interactions (Scholes & Archer, 1997), and facilitate recruitment (Kellman & Miyanishi, 1982; Loudermilk & Cropper, 2007). The presence of trees can affect groundcover biodiversity via pathways such as spatial heterogeneity in fire characteristics and water resources, which can increase the diversity of niche space available to understory plant species and alter the balance of functional groups (Godlee et al., 2021; Platt et al., 2016; Veldman et al., 2013). Trees also play a role in plant–wildlife interactions, such as the dispersal of plant seeds, which may result in local changes to ecosystem structure and function (Abreu et al., 2021; Brewer, 1998). When combined with frequent fires, these processes should reinforce niche selectivity and deterministic assembly of plant communities.

## AUTHOR CONTRIBUTIONS

**Raelene M. Crandall:** Data curation (lead); formal analysis (lead); visualization (lead); writing – original draft (lead); writing – review and editing (lead). **Yingen M. Chew:** Data curation (supporting); formal analysis (supporting); writing – original draft (supporting); writing – review and editing (supporting). **Jennifer M. Fill:** Data curation (supporting); formal analysis (supporting); writing – original draft (supporting); writing – review and editing (supporting). **Jesse K. Kreye:** Conceptualization (equal); writing – review and editing (supporting). **J. Morgan Varner:** Conceptualization (equal); writing – review and editing (equal). **Leda N. Kobziar:** Conceptualization (equal); writing – review and editing (equal).

## FUNDING INFORMATION

Funding was provided by the Joint Fire Science Program (Project 15‐1‐05‐5), the University of Florida Emerging Scholars Program, and the University of Florida Foundation, Inc.

## CONFLICT OF INTEREST STATEMENT

The authors have no conflict of interest to declare.

## Data Availability

The data supporting this study's findings are openly available in Zenodo at https://doi.org/10.5281/zenodo.10161538.
